# Prevalence and epidemiology of *Mycoplasma genitalium* and the absence of macrolide resistance in *M. genitalium* among pregnant women attending antenatal care in Zambia

**DOI:** 10.3389/fpubh.2025.1576376

**Published:** 2025-05-09

**Authors:** Daniel Schröder, Sumire Sorano, Elena Shipitsyna, Enesia Banda Chaponda, Daniel Golparian, Ephraim Chikwanda, Vivian Mwewa, Joyce M. Mulenga, Mike Chaponda, Chris Smith, Matsui Mitsuaki, Massimo Mirandola, Karel Blondeel, Igor Toskin, R. Matthew Chico, Magnus Unemo

**Affiliations:** ^1^Department of Laboratory Medicine, Faculty of Medicine and Health, WHO Collaborating Centre for Gonorrhoea and Other STIs, Örebro University, Örebro, Sweden; ^2^Faculty of Infectious and Tropical Diseases, London School of Hygiene & Tropical Medicine, London, United Kingdom; ^3^School of Tropical Medicine and Global Health, Nagasaki University, Nagasaki, Japan; ^4^Department of Medical Microbiology, D.O. Ott Research Institute of Obstetrics, Gynecology and Reproductive Medicine, St. Petersburg, Russia; ^5^Department of Biological Sciences, University of Zambia, Lusaka, Zambia; ^6^Tropical Diseases Research Centre, Ndola, Zambia; ^7^St. Paul’s Mission Hospital, Nchelenge, Zambia; ^8^Division of Global Health, Department of Public Health, Graduate School of Health Sciences, Kobe University, Kobe, Japan; ^9^Department of Diagnostics and Public Health, University of Verona, Verona, Italy; ^10^Faculty of Medicine and Health Sciences, Ghent University, Ghent, Belgium; ^11^Department of Sexual and Reproductive Health and Research, World Health Organization, Geneva, Switzerland; ^12^Institute for Global Health, University College London (UCL), London, United Kingdom

**Keywords:** *Mycoplasma genitalium*, pregnancy, prevalence, antimicrobial resistance, azithromycin, macrolide resistance, Zambia, antenatal care

## Abstract

**Introduction:**

*Mycoplasma genitalium* (MG) is a sexually transmitted bacterium of public health importance, associated with genitourinary disorders, and adverse reproductive and perinatal outcomes. Global data on MG prevalence and antimicrobial resistance (AMR) are primarily available from high-income countries, whereas there is a dearth of information from resource-constrained settings including sub-Saharan Africa. Furthermore, international data on MG rates and AMR in the antenatal population are scarce. Understanding MG prevalence and AMR patterns is crucial for developing effective public health strategies and treatment guidelines. The aim of this study was to investigate the prevalence and epidemiology of MG and the presence of macrolide resistance-associated mutations (MRAMs) among pregnant women attending antenatal care facilities in Zambia.

**Methods:**

A cross-sectional study was conducted at four antenatal care facilities in Nchelenge, Zambia, among 1,021 pregnant women. Vaginal swabs were collected and tested using the Aptima *Mycoplasma genitalium* assay, Aptima Combo 2 assay and Aptima *Trichomonas vaginalis* assay on the Panther System (Hologic). MG-positive samples were further analyzed for MRAMs using the ResistancePlus™ MG assay (SpeeDx).

**Results:**

The prevalence of MG was 12.6% (127 of 1,005 valid samples) among the pregnant women. Only 12 MG-positive women (9.4%) had symptoms of a genitourinary infection, which was similar to the frequency of genitourinary symptoms among MG-negative women (6.1%). The rates of *Chlamydia trachomatis, Neisseria gonorrhoeae, T. vaginalis*, and HIV seropositivity were 7.4, 8.3, 23.0, and 8.6%, respectively. MG infection was significantly associated with the presence of all other tested sexually transmitted infections and HIV seropositivity: the detection rates of *C. trachomatis, N. gonorrhoeae, T. vaginalis*, and HIV seropositivity were significantly higher in MG-positive than in MG-negative women (15.1% vs. 6.2, 15.0% vs. 7.5, 32.3% vs. 22.0, and 14.3% vs. 7.5%, respectively). The ResistancePlus™ MG assay detected MG in 66.1% (84/127) of samples positive by the Aptima *M. genitalium* assay, however, no MRAMs were detected in the 23S rRNA gene for any of these 84 samples.

**Discussion:**

This study emphasizes the high prevalence of MG among pregnant women in Zambia, but also lack of MRAMs in MG. These findings suggest that azithromycin remains an efficacious treatment option for MG in this population. Nevertheless, continuous surveillance and judicious macrolide use to maintain treatment efficacy are imperative. Further research and sustained monitoring of MG are essential to inform public health strategies and clinical guidelines in Zambia and similar settings worldwide.

## Introduction

The global concern surrounding the prevalence of and antimicrobial resistance (AMR) in *Mycoplasma genitalium* is significant, yet comprehensive data remain lacking worldwide. Most of the available prevalence data for *M. genitalium* and its AMR are derived from high-income regions such as North America (United States and Canada), the European Union (especially Scandinavia and the United Kingdom), Australia, and Japan (1, 2). Accordingly, there is very limited information on *M. genitalium* prevalence and AMR from the World Health Organization (WHO)’s African Region, the Eastern Mediterranean Region, and Latin America and the Caribbean, which belong to the WHO Region of the Americas (1–5).

In women, *M. genitalium* is associated with cervicitis and pelvic inflammatory disease (6, 7), and during pregnancy *M. genitalium* infection is associated with an increased risk of preterm birth (7, 8). In antenatal care, the choice of antimicrobials for *M. genitalium* treatment is limited to macrolides, as fluoroquinolones and tetracyclines, other classes of antimicrobials effective against *M. genitalium*, are not recommended in pregnancy (9). Due to the potential adverse pregnancy outcomes associated with *M. genitalium* infections and the limited choice of treatment options in pregnancy, research into *M. genitalium* prevalence and macrolide resistance-associated mutations in the antenatal population is of great importance.

In the past decade, there has been a marked global increase in macrolide resistance among *M. genitalium* strains (2, 4, 5). The primary mechanism for macrolide resistance in *M. genitalium* is mutations in the 23S rRNA gene, specifically at nucleotide positions A2058 or A2059 (*Escherichia coli* numbering). *M. genitalium* has only one rRNA gene operon and specific mutations in one of these two 23S rRNA gene nucleotide positions changes the azithromycin minimum inhibitory concentration (MIC) value from <0.063 mg/L to >8 mg/L. Consequently, mutations in the 23S rRNA A2058 or A2059 nucleotide positions correlate well with both *in vitro* and clinical azithromycin resistance (4). Many countries worldwide mostly use syndromic management of sexually transmitted infections (STIs) and lack etiological diagnosis of bacterial STIs in general and *M. genitalium*, in particular. This has stymied *M. genitalium* diagnostics and efforts to quantify *M. genitalium* prevalence and AMR data, and hampered development of effective public health strategies and treatment guidelines in response. Gathering detailed global data on the prevalence of *M. genitalium* infection and AMR is crucial for public health (2, 4, 5). Such data are necessary to support evidence-based strategies for the prevention, management, and control of *M. genitalium* infections. Additionally, this information is vital for updating guidelines based on both etiological and syndromic management, ensuring they are informed by the most current and comprehensive evidence. In sub-Saharan Africa, including Zambia, there is an urgent need to understand the local epidemiology of *M. genitalium* and its AMR to inform public health interventions and policy decisions.

The Aptima *M. genitalium* assay (Hologic, San Diego, United States) was the first *M. genitalium* diagnostic test to receive approval from the US FDA. This nucleic acid amplification test (NAAT) has shown a 20–25% increase in sensitivity while maintaining high specificity compared to previously used diagnostic PCR assays (10, 11). The ResistancePlus™ MG (SpeeDx Pty. Ltd., NSW, Australia) is a CE-IVD approved real-time qPCR assay that detects *M. genitalium* by targeting the *mgpB* gene and additionally identifies the five key macrolide resistance-associated mutations in the 23S rRNA gene: A2058G, A2059G, A2058C, A2059C, and A2058T (*E. coli* numbering).

The main aim of the present study was to investigate the prevalence and epidemiology of *M. genitalium* among pregnant women attending antenatal care facilities in Zambia using the Aptima *M. genitalium* assay on the Panther Platform (Hologic). Additionally, the prevalence of macrolide resistance-associated mutations in *M. genitalium*-positive samples was examined using the ResistancePlus™ MG real-time qPCR assay (SpeeDx). Accordingly, this study aimed to provide critical data on *M. genitalium* infection and macrolide resistance patterns, which are essential for developing effective public health strategies and updating clinical guidelines. This research is particularly important in the antenatal care setting, where timely and accurate diagnosis and treatment of *M. genitalium* can significantly impact maternal and neonatal health outcomes.

## Materials and methods

### Study population and specimen collection

This was a nested study that was part of a larger evaluation of point-of-care tests for *Trichomonas vaginalis* and bacterial vaginosis registered in the Pan African Clinical Trials Registry (PACTR202302766902029) and conducted from 15 February to 26 May 2023 at four antenatal care facilities in Nchelenge, Zambia: Kabuta, Kafutuma, Kashikishi, and Nchelenge. Pregnant women attending these facilities were recruited based on the following inclusion criteria: (i) at least 13 weeks of gestation; (ii) no self-reported use of metronidazole or clindamycin during the current pregnancy; (iii) no known allergies or contraindications to metronidazole; and (iv) no use of vaginal creams or ointments, douching, or vaginal lubricants within 72 h prior to recruitment. These inclusion criteria were designed for the initial *T. vaginalis* study, results of which have been published elsewhere (12). Written informed consent was obtained from all participants before specimen collection. Vaginal swabs were collected from each participant using the Aptima Vaginal Swab Specimen Collection kit (Hologic). Background and clinical information including self-reported genitourinary symptoms (for example, unusual vaginal discharge, pain during urination, itching or burning of the vulva) was collected from all consenting participants.

### Detection of *Mycoplasma genitalium* and other STIs

The Aptima *M. genitalium* assay, Aptima Combo 2 assay and Aptima *T. vaginalis* assay on the Panther System were used to detect *M. genitalium*, *C. trachomatis* plus *N. gonorrhoeae* and *T. vaginalis*, respectively, in full concordance with the manufacturer’s instructions. Determine® HIV Test Kit (Abbott, Illinois, United States) was used for HIV screening.

### Detection of macrolide resistance-associated mutations

Samples positive for *M. genitalium* using the Aptima *M. genitalium* assay were further analyzed for macrolide resistance-associated mutations using the ResistancePlus™ MG assay (SpeeDx).

### Statistics

All numerical variables were ranked as having non-normal distribution using the Shapiro–Wilk test and were presented by medians with interquartile range (IQR), with differences between the groups computed using the Mann–Whitney test. Categorical variables were presented by percentages and prevalence rates were reported with 95% confidence intervals (95% CIs). The differences in the frequencies of *M. genitalium* associates between *M. genitalium*-positive and *M. genitalium*-negative women were evaluated using Pearson’s chi-square or Fisher’s exact (whenever appropriate) tests. Statistical analyses were performed with the use of the statistics package IBM SPSS Statistics 27 (IBM). All tests for significance were two-sided, and statistically significant differences were assumed when *p* < 0.05.

## Results

### Prevalence and epidemiology of *Mycoplasma genitalium*

A total of 1,021 women were enrolled in the study; 16 samples were reported by the Panther System as invalid in the Aptima *M. genitalium* assay. Of the 1,005 valid vaginal swab samples, 127 (12.6% [95% CI 10.7–14.8%]) tested positive for *M. genitalium* using the Aptima *M. genitalium* assay (Table 1).

**Table 1:** Prevalence of *Mycoplasma genitalium* (MG) and macrolide resistance-associated mutations in women attending antenatal care in Zambia.

Population/specimen	Prevalence [95% CI] (No. of MG positive tests/no. of valid tests)
Positive samples with Aptima MG assay	12.6% [10.7–14.8%] (127/1005)
Positive samples with Aptima MG assay and ResistancePlus™ MG assay	66.1% (84/127)
23S rRNA gene mutations	0% [0–5.3%] (0/84)

CI, confidence interval; No., number.

Women with *M. genitalium* infection were significantly younger (median, 22 years; IQR, 19–25 years) than *M. genitalium*-negative women (median, 23 years; IQR, 20–30 years; *p* = 0.015). Gestational age of *M. genitalium*-positive women (median, 25 weeks; IQR, 21–31 weeks) did not differ from that of *M. genitalium*-negative women (median, 26 weeks; IQR, 21–32 weeks; *p* = 0.458). Only 12 women (9.4%) infected with *M. genitalium* had symptoms of a genitourinary infection, which did not significantly differ from the frequency of genitourinary symptoms among *M. genitalium*-negative women (6.1%; Table 2).

**Table 2:** *Mycoplasma genitalium* (MG) correlates in women attending antenatal care in Zambia.

	Aptima MG-positive women	Aptima MG-negative women	*p*-value
	No. of positive samples/total no. of valid samples (%)		
Genitourinary symptoms^a^	12/127 (9.4%)	53/874 (6.1%)	0.148
*Chlamydia trachomatis*	19/126 (15.1%)	54/871 (6.2%)	**<0.001**
*Neisseria gonorrhoeae*	19/127 (15.0%)	65/870 (7.5%)	**0.005**
*Trichomonas vaginalis*	41/127 (32.3%)	192/873 (22.0%)	**0.010**
Any STI	59/127 (46.5%)	252/873 (28.9%)	**<0.001**
HIV seropositivity	18/126 (14.3%)	65/865 (7.5%)	**0.010**

Significant *p*-values are shown in bold letters.

a Dysuria and/or itching and/or vaginal discharge.

The frequencies of *C. trachomatis, N. gonorrhoeae, T. vaginalis*, and HIV seropositivity were 7.4% [95% CI 6.0–9.2%] (75/1011), 8.3% [95% CI 6.8–10.2%] (84/1011), 23.0% [95% CI 20.5–25.6%] (233/1015), and 8.6% [95% CI 7.0–10.5%] (87/1011), respectively. *M. genitalium* infection was significantly associated with presence of all the other tested STIs: the detection rates of *C. trachomatis, N. gonorrhoeae, T. vaginalis*, and any of the three STIs were significantly higher in *M. genitalium*-positive than in *M. genitalium*-negative women (15.1% vs. 6.2, 15.0% vs. 7.5, 32.3% vs. 22.0, and 46.5% vs. 28.9%, respectively; Table 2). There was also a significant association between *M. genitalium* infection and HIV seropositivity (14.3% vs. 7.5%; Table 2).

### Detection of macrolide resistance-associated mutations

The ResistancePlus™ MG assay detected MG in 66.1% (84/127) of samples positive by the Aptima *M. genitalium* assay. However, none of these 84 *M. genitalium*-positive samples exhibited any macrolide resistance-associated mutations (A2058G, A2059G, A2058C, A2059C, and A2058T) in the 23S rRNA gene (Table 1).

## Discussion

The present study on the prevalence and epidemiology of *M. genitalium* and its lack of macrolide resistance among pregnant women attending antenatal care facilities in Zambia contributes valuable data to the limited information available from the WHO African Region. A high prevalence (12.6%) of *M. genitalium* was identified in this population in Zambia, but no macrolide resistance-associated mutations were detected in any *M. genitalium-*positive sample. This lack of macrolide resistance in *M. genitalium* stands in contrast to the rising global trends of high prevalence of macrolide resistance-associated mutations in *M. genitalium*-positive samples, especially in regions such as North America, Europe, Australia, and Japan (2, 4, 5).

Globally, *M. genitalium* prevalence and AMR data are predominantly available from high-income countries with significant gaps in data from resource-limited settings such as sub-Saharan Africa (2, 4, 5). Studies in high-risk populations across multiple countries have shown *M. genitalium* prevalence rates ranging from 10% to over 20%, with rates of macrolide resistance-associated mutations frequently exceeding 40% in several regions (1, 2, 4, 5). For instance, a recent study detected *M. genitalium* in 19.1% of women at high risk of STIs (12.4% in Guatemala, 16.0% Morocco, 22.1% South Africa) (13). The prevalence of macrolide resistance-associated mutations among women at risk in South Africa was found to be relatively low (2.4%). In contrast, other regions showed higher resistance rates: Morocco had 11.6% macrolide resistance-associated mutations and Guatemala 4.8% (13). These variations highlight the significant geographical differences in *M. genitalium* prevalence and AMR patterns, underscoring the need for region-specific public health strategies and continuous surveillance to effectively manage *M. genitalium* infections.

Because most research efforts regarding *M. genitalium* prevalence and AMR are focused on high-risk populations, there are limited data in lower-risk patients, such as pregnant women. In the antenatal population, the prevalence of *M. genitalium* is estimated to be around 1% in high-income settings, similar to that in the general population (1). According to one study, frequency of macrolide resistance-associated mutations in antenatal care in high-income settings may be as high as 30% (14). In low- and middle-income countries, *M. genitalium* prevalences of 3.6 to 12.5% have been reported among pregnant women in South Africa and Papua New Guinea (15–19), which aligns with this study. We also found no macrolide resistance-associated mutations in the antenatal population in Zambia as reported in several studies from South Africa and Papua New Guinea (16, 18, 20). However, one study from 2022 examining South African pregnant women found a single *M. genitalium*-positive sample (1 of 14 samples) with macrolide resistance-associated mutations (23S rRNA A2059G mutation) (17). The absence of detected macrolide resistance-associated mutations among *M. genitalium-*positive samples in our study population is a critical finding, which may be associated with the lack of etiological STI screening and subsequent treatment of asymptomatic *M. genitalium* infections in Zambia. It suggests that unlike in many other regions, the use of azithromycin for treating *M. genitalium* infections in Zambia may remain effective. This is in sharp contrast to findings from regions where macrolide resistance is high, and alternative treatments such as moxifloxacin are often recommended (2–4). Notably, in Zambia the first-line syndromic treatment for vaginal and urethral discharge is ceftriaxone 500 mg plus azithromycin 1 g plus metronidazole 2 g.^1^ However, international guidelines on the management of *M. genitalium* infections recommend a first-line treatment including azithromycin 1.5–2.5 g taken over 3–5 days, that is, if no macrolide resistance has been detected. These extended azithromycin dose regimens have a higher efficacy than the azithromycin 1 g single dose and also reduce the risk of azithromycin resistance emergence. Furthermore, in several of these international guidelines the azithromycin treatment is preceded by a doxycycline regimen (100 mg twice daily for 7 days). In Zambia and elsewhere, the use of azithromycin for treatment of STIs should be approached with caution and for the treatment of *M. genitalium* infections, azithromycin 1 g single dose therapy should ideally not be used (4). The global increase in macrolide resistance in *M. genitalium* and other STIs is alarming and underscores the importance of ongoing surveillance and judicious antibiotic stewardship. Maximizing the useful therapeutic life of azithromycin requires stringent measures to ensure proper diagnosis before treatment and prevent its overuse.

Our results align with global data from previous studies (1, 2, 4, 5, 10), which emphasize significant geographical variation in *M. genitalium* prevalence and AMR patterns. While our study did not detect any macrolide resistance, the high prevalence of *M. genitalium* shows a substantial burden of *M. genitalium* infections (many with concomitant other STIs) among pregnant women in Zambia, necessitating attention to both screening and treatment protocols. These findings underscore the importance of regional surveillance to identify and address specific public health needs with the global aim of filling gaps in data from sub-Saharan Africa.

The results of our study confirm that *M. genitalium* infection in women is mostly asymptomatic, i.e., only 9.4% of the *M. genitalium*-positive women had genitourinary symptoms, which was similar to the frequency of genitourinary symptoms among *M. genitalium*-negative women (6.1%). Furthermore, high rates of other STIs and HIV seropositivity (7.4%, 8.3%, 23.0%, 8.6% for *C. trachomatis*, *N. gonorrhoeae, T. vaginalis,* and HIV seropositivity, respectively), as well as strong associations of *M. genitalium* with other STIs and HIV seropositivity were observed among pregnant women in Zambia. Nearly half of the *M. genitalium*-positive women had one or more of the other three STIs. Our results support recent findings from Papua New Guinea (18) where around 40% of *M. genitalium*-positive pregnant women had one or more concurrent STIs. In resource-constrained settings, etiological diagnosis of STIs remains difficult, and syndromic management is mostly used. The limitations of syndromic management for STIs, as highlighted by Wi et al. (21), underscore the importance of integrating accurate and affordable NAATs (laboratory-based and at point-of-care) for detection of *M. genitalium* and other STIs into public health strategies, including antenatal care. This approach can significantly improve the diagnosis and treatment of *M. genitalium* and other STIs, thereby reducing the rates of overtreatment and missed diagnoses.

The cross-sectional design of our study provides a snapshot of the current prevalence and AMR patterns but does not account for temporal changes. Longitudinal studies are needed to monitor trends over time and assess the impact of interventions aimed at reducing *M. genitalium* prevalence and AMR. Such studies will help understand the dynamics of *M. genitalium* infections and AMR development. Nevertheless, our findings highlight several key recommendations for public health practice in Zambia and similar settings. Continuous monitoring of *M. genitalium* prevalence and AMR patterns is essential, and establishing robust surveillance systems will provide timely data to guide treatment protocols and public health interventions. Judicious use of antibiotics, particularly azithromycin, is critical to prevent the development of AMR. This requires ensuring accurate diagnosis and appropriate use of antibiotics. With the lack of appropriate natural history studies estimating the risk of sequelae subsequent to asymptomatic infections, the benefits of treating many asymptomatic infections have been considered to be outweighed by the risk of increased AMR from widespread treatment (3, 4). Appropriate education of healthcare providers and the public about *M. genitalium* and its potential implications for reproductive health is crucial; awareness campaigns can help reduce the stigma associated with STIs and promote timely healthcare-seeking behaviors.

In conclusion, the 12.6% prevalence of *M. genitalium* among pregnant women in Zambia, coupled with the absence of azithromycin resistance-associated mutations, provides critical insights into the epidemiology of *M. genitalium* in Zambia and the sub-Saharan African region. These findings underscore the need for targeted public health strategies to manage *M. genitalium* infections effectively and prevent the emergence of resistance. Further research and sustained surveillance efforts are essential to safeguard the reproductive health of women in Zambia and similar settings. Additionally, it is crucial to, as feasible, replace the syndromic STI management with etiological diagnosis of non-viral STIs in these settings.

## Data Availability

The original contributions presented in the study are included in the article/supplementary material, further inquiries can be directed to the corresponding author.

## Footnotes

^1^ https://www.differentiatedservicedelivery.org/wp-content/uploads/August-2022-Zambia-Consolidated-Guidelines.pdf
